# Collaborative research protocol to define *patient-reported experience measures* of the cystic fibrosis care pathway in France: the ExPaParM study

**DOI:** 10.1186/s13023-022-02204-0

**Published:** 2022-02-22

**Authors:** D. Pougheon Bertrand, A. Fanchini, P. Lombrail, G. Rault, A. Chansard, N. Le Breton, C. Frenod, F. Milon, C. Heymes-Royer, D. Segretain, M. Silber, S. Therouanne, J. Haesebaert, C. Llerena, P. Michel, Q. Reynaud

**Affiliations:** 1 Laboratory of Education and Health Practices (LEPS) UR3412, Sorbonne Paris Nord University, Villetaneuse, France; 2 Centre de Ressources et de Compétences mucoviscidose, Hôpital Couple-Enfants, Grenoble, France; 3 Laboratory RESHAPE U. INSERM 1290, Claude Bernard Lyon 1 University, Villeurbanne, France; 4 Quality and Security Department, Hospices Civils de Lyon, Lyon, France; 5 Centre de Ressources et de Compétences mucoviscidose, Hôpital Lyon Sud, Pierre-Bénite, France; 6 Centre de Ressources et de Compétences mucoviscidose, CHU Lille, Lille, France; 7 Cystic Fibrosis Patient and Parent Co-Investigators Group, Paris, France

**Keywords:** Collaborative research, Patient partnership, Patient experience, Patient-reported experience measures, Cystic fibrosis care, Quality improvement

## Abstract

**Introduction:**

In France, the cystic fibrosis (CF) care pathway is coordinated by multidisciplinary teams from specialised CF centres or transplant centres. It includes the care provided at home or out of hospital, risk prevention in daily life and adjustments to social life, which together contribute to the person’s quality of life. Patient experience is used to describe and evaluate the care and life of patients living with the disease.

**Objectives:**

Our collaborative research aims to identify the most significant areas and criteria that characterise the CF pathway. It will lead to the development of a questionnaire to collect patients' experience, which can be administered to all patients or parents of children registered and followed in the centres. The article describes the protocol developed in partnership with patients and parents of children living with the disease.

**Method:**

A multidisciplinary research group brings together researchers, patients, parents of children with CF and health care professionals. The patient partnership is involved in the 4 phases of the protocol: (1) setting up the study, recruiting patient and parent co-researchers, training them in qualitative research methods, defining the situations and profiles of patients in the study population, elaborating the protocol; (2) selecting the study sites, recruiting participants, carrying out semi-structured interviews, analysing verbatims using the grounded theory approach; (3) co-elaborating *Patient-Reported Experience Measures* (PREM) questionnaires adapted to the 4 types of participants: parents, adolescents, non-transplanted adults and transplanted adults; (4) validating the construct with participants and professionals from the study centres.

**Results:**

The protocol obtained a favourable opinion from the Ethics Evaluation Committee of INSERM (IRB00003888—no. 20-700). Training was provided to the 5 patients and 2 parent co-researchers to enable them to participate effectively in the research. Eleven centres participated in the recruitment of participants in mainland France and Reunion Island. Eighty hours of interviews were conducted.

**Discussion:**

The PREM questionnaires to be elaborated will have to undergo psychometric validation before being used by the actors of the CF network to assess the impact on the care pathways of quality approaches or new therapies available in cystic fibrosis.

*Trial Registration Registry*: IRB00003888 – no. 20-700. Issue date: 06/09/2020.

## Introduction/background

Patient experience (PE) has increasingly been taken into account in recent years to evaluate the quality of care through the experiences reported by patients, in addition to patient satisfaction surveys [1–3]. Patient experience is a concept that originates from the USA, promoted by the Beryl Institute [4] and widely disseminated by the Patient Experience Journal [5]. PE is defined as all the interactions and situations experienced by a person or their family or caregivers during the course of their care. These interactions are shaped both by the organisation of this care pathway and by the person's life history (definition from the French Patient Experience Institute adapted from the Beryl Institute's [6]). This definition is based on the sum of interactions between the patients and their health care system, according to the organisation of their care pathway, and combines an objective approach based on facts and a subjective approach of their experience in these different circumstances. While one of the objectives of hospital care is to improve patient experience, it is a challenging task since experience is subjective, not always rational, and influenced by multiple factors at an individual level [7]. In order to make this concept operational, PE questionnaires were developed to obtain results that can be acted upon, with the aim of improving the quality and safety of care. These questionnaires, named PREM (patient-reported experience measures), are intended to be completed by patients without the intermediation of health professionals [8]. They can be used to improve the quality of services within a health care organisation, to compare results between several organisations (benchmarking) or to allocate resources to certain organisations, services or professionals (pay for performance) according to the results observed. Depending on the objectives set, generic questionnaires (quality of hospital catering services, evaluation of hygiene measures or reception on arrival in the establishment) or questionnaires that are specific to certain care pathways or pathologies are used. The Picker Institute Europe database offers validated questionnaires covering seven general themes (sharing of information, coordination of care, physical comfort during hospitalisation, emotional support and respect, respect for patients’ preferences, involvement of friends and family, continuity of care) as well as tools specific to certain pathways such as the transition to adult care or pathologies such as sickle-cell anaemia [9].

However, various criticisms have emerged about these tools, raising questions about the process of constructing PE surveys, their sensitivity, and the degree to which they are used by stakeholders to evaluate and guide measures to improve the quality of care [10–12]. Specific questions are added in the case of chronic diseases. *First*, for patients living with a chronic disease, PE develops over the course of their journey with the disease, which is seen as the patients' pathways of care, health, and life in their social environment [13]. This pathway begins around the time of diagnosis and when the disease is announced to the patient, and it includes the successive management by different actors and in different health care or medico-social establishments or structures. However, diagnosis is not always the beginning of this pathway, especially in the case of rare diseases in which situations of diagnostic wandering can be an integral part of the person's care and life pathways. Patient experience is also contributed to by that of their family members, particularly those who "naturally" take on the role of caregivers and participate in improving their quality of life. However, the tools used to collect PE rarely take into account all events along this pathway, nor the patients’ quality of life, their university studies or working conditions, but rather particular episodes that are considered to be critical (surgery and postoperative rehabilitation) or subject to a priority care policy (paediatric to adult care transition). This causes the aspects that are ignored to remain unnoticed and PE to be underused to improve patients’ pathways. *Second*, criticisms point to the lack of involvement of patients or their representatives in the development of PE collection tools, which was echoed in the report “Being a Patient” published by The Patients' Association [14]. Ways to better take into account the needs of patients in the evaluation of their experience were suggested, such as: including social needs beyond care and focusing on the patient's life with the disease; focusing on the patient's experience rather than on the service provided by health care providers; using new methodologies to discover new aspects of the patient's experience; taking into account the impact of the disease on patient health outcomes when evaluating their experience. *Third*, several publications question the use of the results and the actual usefulness of Patient Experience Surveys to improve the quality of services provided to patients [15]. The authors emphasise the need for a clear understanding of patient outcomes and of expertise in quality improvement approaches to achieve service improvement. The availability of PE results is not sufficient to drive the changes needed to improve services. The necessary conditions to implement quality improvement initiatives successfully have been widely identified, including the culture of the organisation, leadership style and patients’ level of engagement [16, 17]. An extensive study conducted by the DUQUE consortium showed that institutional quality management strategies have little impact on generic PREM scores across various European countries [18]. The reasons are diverse: quality management strategies may have only been partially implemented; these strategies may not directly affect PE, which could be more sensitive to direct patient-clinician interactions, or due to the fact that not all patients would benefit from them; and, more fundamentally, this “*loose coupling may reflect a situation where hospitals created a 'facade' of quality management strategies to attract recognition, funding, patients, and status, while not successfully pursuing their implementation*” [19].

Our research takes place in a rare disease network in France that is structured around paediatric, adult or mixed centres and transplantation centres for lung transplant patients. This study is the continuation of a collaborative approach to improve the quality of care carried out between 2011 and 2019, replicated in France from the *Quality Improvement Program (QIP)* deployed by the Cystic Fibrosis Foundation in the USA [20]. This QIP involved patients and parents of children with CF from diagnosis of the organisation to the implementation and evaluation of improvement actions at their centre [21]. The involvement of patients and parents was evaluated in terms of its perceived usefulness in improving care [22]. The patient and parent partners [23] have suggested that the experience of patients followed in the centres be collected extensively, to ensure that the difficulties encountered by socially disadvantaged groups are taken into account to ensure better representativeness and equity. These suggestions highlighted the need for an instrument that can both describe the experience of care and life pathways and be sensitive to the improvement actions implemented. Several countries have developed cystic fibrosis patient experience questionnaires [24, 25] but it appears that they focus mainly on hospital care, without taking into account the care provided outside of the hospital and at home, because their care models are centred on specialised centres. Consequently, the questionnaires do not cover the daily living conditions with the disease, or only to a limited extent. The aim of our study is to create a PREM to get a better understanding of the relevant actions required to improve the care and lives of the greatest number of the people living with CF in France. Comparisons with international surveys will be conducted during the study and a collaboration with the CF Foundation will be organised to benefit from their expertise of the development and use of a CF PREM.

## Methods

### Collaborative research

The study design is that of collaborative research, involving representatives of people living with CF, together with care professionals, in the development of PREMs. Collaborative research offers a model for studying societal issues by bringing together academic researchers and professionals who act on these issues, with patients when it is related to health matters [26]. Increased levels of participation among patients enhance the relevance and quality of the results, while the participation of professionals enhances the uptake, sustainability, and transferability of improvement programmes developed as a result of the research [27]. To enhance the ability of the patients and parents to participate effectively in all stages of the research, training in the use of qualitative methodology and tools will be provided by the academic researchers. The modalities of collaboration between patients and researchers during the collection and analysis of the data, and the study process will be documented to guarantee the quality and scientificity of the research [28, 29]. Finally, the results will be checked with the study population in order to ensure their reliability and to validate the construct, beyond its co-construction with the co-researchers.

### Description of the CF pathway

**Fig. 1:**
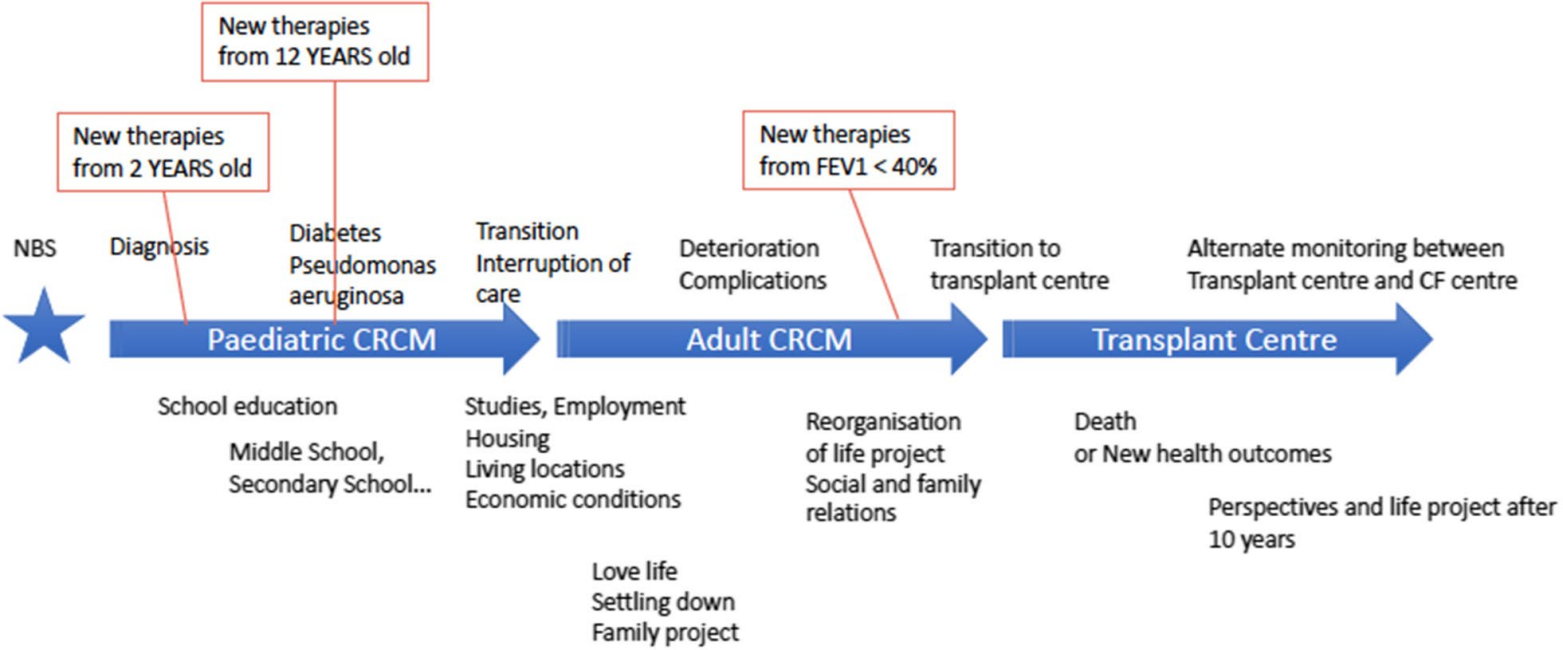
Overview of the cystic fibrosis pathway from neonatal screening to post-transplant care

The methodology is based on a descriptive view of the current pathway (Fig. 1). The experience of the clinicians, patients, and parents in the research group is decisive in identifying the situations to be investigated in the questionnaire. Four groups have been identified along the cystic fibrosis pathway: parents of children with cystic fibrosis from birth (median age at diagnosis: 1.1 months) up to 15 years of age, adolescents between 16 and 18 years of age followed in a paediatric CF centre, thus preparing their transition to adult care, non-transplanted adult patients followed in an adult CF centre and transplanted adult patients followed in a transplant centre for post-transplant care. Therefore, our study does not constitute a longitudinal analysis of biographical patient pathways. The cross-sectional approach is consistent with the presentation of the health outcomes in the French CF Registry, which also reflects the therapeutic progress and changes in the conditions of care for this disease over the last 40 years. In order to reflect the current living conditions with cystic fibrosis and conditions of care, the period chosen for the patient and parent survey is the last 18 months, complemented by the experience of transitions that have occurred in the last 5 years (Diagnosis, Adolescent-Adult care transition, Transition to transplant).

### Grounded theory approach

This study is conducted using the inductive approach of grounded theory [30] without pre-established theory, to develop a PREM tool that reflects a wide variety of situations of care and life with cystic fibrosis in France (metropolitan and overseas departments). With Saunders [31], we clarify some of the theoretical and practical implications of this objective:

1. As the study sample is designed to reflect the variety of situations, it is not meant to be representative of these situations in proportion to their frequency in the general CF population: “unconventional pathways” will be included, such as that of a family residing in French Guyana and followed in a CF centre in Paris, or a migration pathway of a family with a child with CF coming from North Africa or a transplant pathway between Reunion Island and metropolitan France. The number of individuals in the sample will be decided on by cross-checking the different pathways with socioeconomic, cultural, and geographic criteria in order to reflect the variety of situations as accurately as possible, and according to whether the recruitment of participants is feasible.
2. Data saturation will apply to both the collection and analysis:
  During interviews, saturation will be reached when the interviewer feels they have a full understanding of the participant's perspective [32].
  During the analysis, saturation will be reached through the stabilisation of the codebook and categories, as well as through the variety of codes within categories [33]. The “unconventional cases” will enrich the list and definitions of categories with the widest possible range of data.

Additional inclusions may be necessary for certain situations, if patients decline to participate, if some interviews are not informative enough, or if there are too few examples in each category to identify the characteristics of concepts [34]. The decision to add to the sample or to merge categories will be taken in agreement with the researchers depending on the data collection and analysis, which will be carried out simultaneously. In this approach, the number of interviews estimated per participant profile is not meant to reiterate the same information but to show the variety of situations along the cystic fibrosis pathway and the richness of the categories (domains and subdomains) of the patient experience.

### Main objective of the study

- Defining a questionnaire to collect patient experience along the cystic fibrosis pathway (PREM) in France and the methods for its administration.

### Secondary objectives of the study

- Identifying domains and quality standards to evaluate the cystic fibrosis pathway, based on the experience of the patients and parents;
- Highlighting the differences between the patient experience questionnaire (PREM) developed during the study and the international questionnaires available for this condition.

### Study design

The research is based on a qualitative approach [35] in four phases.

#### PHASE 1: Setting up the research and finalising the protocol

*Creating a multidisciplinary group of co-researchers* [36] Involving health professionals, researchers, patients, and parents of children with CF, all of whom are referred to as co-researchers contributing to the validation of protocol tools, submission to ethics committee, data analysis methods, synthesis of the results, elaboration of PREM questionnaire, valorisation of research. Patients and parents were recruited with the association Vaincre la Mucoviscidose through a call for interest on social media and selected based on their CV and a motivation letter, and following an interview with academic researchers. Compensation for the time spent on the project is provided in addition to the reimbursement of their travel expenses.

*Training of the patient/parent co-researchers* in the characteristics, methods, and instruments of qualitative research by the academic researchers from the LEPS/USPN.

During this training, theoretical learning is combined with practical application to the context of the research, in order to write the protocol and define the data collection tools. Notably, the different *situations* lived by CF patients or parents along the CF pathway are specified in order to be investigated during the research. They are defined using different variables: health status, age, transplant status, sociocultural determinants, where they live (mainland France and overseas departments, urban settings, or countryside), and type of centre. They are then translated into participant *profiles* (Table 1) complemented by the necessary *family, socio-professional and geographic criteria* (Table 2).

**Table 1:** List of profiles

Paediatric
P1 = patient between 0 and 5 years
P2 = patient between 6 and 11 years
P3 = patient between 12 and 15 years
P4 = patient > 15 years and < 18 years (adolescents)
P5 = patient who has changed CF centre in the last two years
P6 = patient who received a CFTR modulator/potentiator
P7 = minor patient diagnosed with COVID-19
P8 = minor patient in the paediatric transplant process
*Adults*
P10 = patient who came to the adult centre following transition of care between 18 and 22 years of age
P11 = patient with late diagnosis in the last 4 years
P12 = 22–26-year-old patient stabilised at CF centre
P13 = patient with complications and progression of disease severity
P14 = patient travelling abroad for > 3 months in the last 3 years
P15 = patient planning to have a child or in the process of MAP
P16 = patient with children
P17 = patient who received a CFTR modulator/potentiator
P18 = adult patient diagnosed with COVID-19
P19 = patient without major complications following transplant
P20 = patient with long-term complications following transplant

**Table 2:**
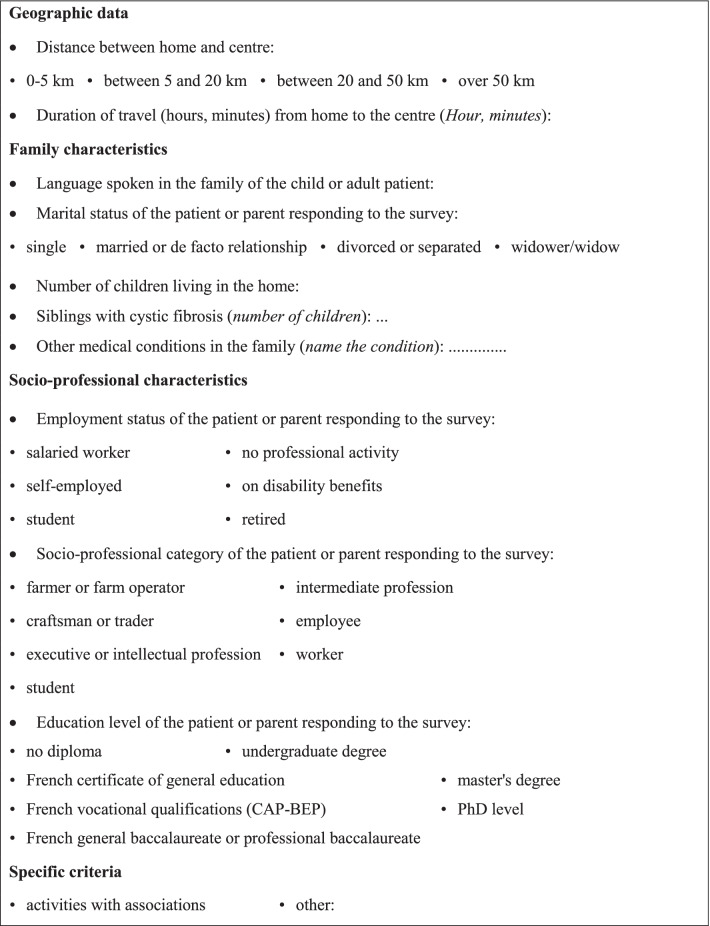
Additional criteria for the patient and parent sample

*Drafting of the detailed study protocol by the group of researchers and co-researchers* for submission to and approval by the INSERM ethics review committee.

#### PHASE 2: Collecting patient experience from study participants

*Selecting the study sites* CF centres have been selected based on a general agreement among the clinicians of the research group who considered that their patient population could allow the recruitment of patients who fit in the different demographic and socioeconomic profiles and criteria that were defined, and that the centre directors would accept to participate in the study.

*Recruiting the study population* the recruitment process was explained to the physician and a healthcare provider in each associated centre during two videoconferences, one on paediatric care and one on adult care. Each physician proposes a list of eligible patients by profile, by filling in the data relating to the criteria for each patient likely to participate (Appendix 1). Upon receipt of all the lists, the scientific coordinator proceeds to a harmonisation in order to ensure the representation of the various profiles. Recruitment of the patients is conducted by the partner centres, following the list adjusted by the coordinator. The individuals who are proposed but not selected on the national list are kept as potential participants who may be included later on to replace a patient failing to be contacted or to enrich the description of the categories in the course of the analysis of the data. Once a participant is included by a centre, their contact details are transmitted to the scientific coordinator to schedule the interview. The participants are asked to take part in a semi-directive interview lasting about 1.5 h (phase 2) and to test a questionnaire (phase 3). Their consent to participate in the study is obtained at the beginning of the recording of the interview. No compensation is planned for study participants.

*Collecting data from patient experience* during interviews conducted by researchers and co-researchers and transcribing their verbatim. Following the withdrawal of a few participants, the recruitment can be completed using the complementary lists in the centres in order to ensure the diversity of situations investigated and the data saturation [37].

*Data analysis using an inductive approach* [38] in the qualitative analysis of interviews, using the grounded theory approach, conducted jointly by the academic researchers and co-researchers [39]. The coding of each interview using NVivo® by a researcher allows the identification and aggregation of units of meaning. The codebook and categories result in the characterisation of domains and criteria of PE. The results are shared at a meeting attended by the entire research group, including care professionals, to reflect on the domains of patient experience related to care and to living with the disease, as well as the main transitions in the pathway.

*Putting the results into perspective* with the domains and items of the *Patient and Family Experience of Care* survey conducted in the USA in order to examine the similarities and differences with the domains and criteria highlighted by our research, which may be related to differences in care, health care systems, societal norms or study methodology.

#### PHASE 3: Development of the questionnaire to collect patient experience

*Development of the PREM questionnaire* based on the domains and criteria described in Phase 2. The criteria of PE in the different domains are broken down into questions with a suitable response format (free response, response scale for each item: 4-degree Likert scale, or a predefined list of answer options). A customisation of the questionnaire allows the targeting of each of the 4 audiences (parents, adolescents, non-transplanted adult patients and transplanted adult patients) either at item level (questions) or in the answers suggested. The questionnaire for each targeted audience is tested and amended with patient and parent co-researchers to ensure that the terms and response formats used are relevant and comprehensible. Ethics approval of the questionnaire and its administration process must be granted before it is sent to the participants.

*Completion of the online questionnaire* developed with Lime Survey sent by email to the participants. The data collected is analysed by the research group and a synthesis of the results made available to the CF centres to prepare the evaluation of construct validity.

*Putting the questionnaire into perspective with other questionnaires used* in the USA and in Europe [25, 26] to consider the integration of common scores by domains of care such as: a score for the *control of cross-infections* (**Infection Control**) during hospital visits, a score for *collaboration between the patient or parent and health care team* (**CollaboRate**) [40], a *coordination score between caregivers* (**IntegRate**) and a *global care score* (**HolisticRate**) including patients’ or parents’ psychological and social-economic aspects.

#### PHASE 4: Construct validity of the questionnaire

This phase aims to establish the construct validity [41] of the questionnaire:

1. for patients: usefulness in evaluating their own patient pathway in all its various dimensions, by means of specific questions at the end of the questionnaire;
2. for the care teams and the QI coordinators in the CF centres: usefulness in understanding the main elements of patient experience of the cystic fibrosis pathway and the usefulness of the results to improve the quality of care and services for patients, by means of videoconferences;

Psychometric validation of the questionnaire is not performed in this study.

### Study population

The number of patients to be included was estimated by patient profile (P1 to P20) using purposive sampling to achieve both representativeness of the diversity of situations and sufficient saturation of codes and categories during the inductive analysis.

The number of targeted inclusions is 57 participants resulting from the consolidation of the numbers in the profiles (Px). In each profile, the number of individuals reflects the demographic, socioeconomic status, and geographic criteria that must be represented in the sample (Table 2). These recruitment objectives are communicated to CF centres in order that they search through their patient population for individuals who may correspond to them:

- 20 parents: 7 parents of children < 5 years old (P1), 7 parents of children between 6 and 11 years old (P2), 6 parents of children between 12 and 15 years old (P3),
- 6 adolescents between 16 and 18 years old (P4),
- 19 non-transplanted adult patients: 6 patients between 18 and 22 years old (P10), 2 patients with late diagnosis (P11), 6 patients between 23 and 30 years stabilised at the CF centre (P12), 5 patients having evolved to a severe state (P13)
- 12 transplanted adult patients: 6 patients without major complications (P19) and 6 patients with major post-transplant complications (P20).

Patients who cannot be interviewed in English or French are excluded from the study.

### Interview guide

The interview guide is structured in four parts:

- *an introductory part* is common to all participants to remind the main information on the study, the time period for the experience to be related, the rules of confidentiality, and the recording procedures;
- *a section on cystic fibrosis care and treatment*, including care at the CF centre, at home, during hospital stays, and out of hospital, the burden of care carried out by the parent or patient at home or out-of-hospital, psychological needs and support, experience with a CFTR modulator/potentiator therapy, and pathway to procreation for adults;
- *a section on life with cystic fibrosis*, including work or study conditions, financial and social situation, changes in living environment, housing conditions, social relations, possible experiences of living abroad, experience of research and new therapies;
- *a section on the transitional phase* to their current stage of the CF pathway: the period around the child's diagnosis of CF, the period of arrival at the adult CF centre, and the transition to transplant care.

The guide includes open-ended and follow-up questions adapted to the profiles of the respondents, based on the characteristics of health outcomes and complications, as reported in the French Registry.

### Qualitative analysis

The interviews are transcribed and analysed using N'Vivo® in an iterative manner, according to a coding framework that emerges during the process of analysis, differently for each participant group: parent, adolescent, non-transplanted adult patient and transplanted adult patient. Coding is done by the researchers in collaboration with co-researchers. The coding framework established in NVivo® is articulated in categories, called domains, grouping subdomains of care and life with CF, which emerge from the interviews [39]. Each category is described with its properties (what it is composed of: subdomains), the conditions of its existence, its various possible forms and dimensions. Relations between categories may appear: for example, a change in parents’ social lives and family relations due to the dietary recommendations and hygiene precautions given at the diagnosis of the child's disease; or a change in the lives of relatives due to the recovery of the patient’s physical capacities after lung transplantation, as they no longer need the assistance they required when waiting for a transplant [39].

### Development and construct validation of the French CF PREM

The development of the questionnaire is based on the domains identified (N'Vivo® categories) by breaking them down into questions. For example, the "*Continuity of remote patient care*" domain can be broken down into: What are the reasons for contacting the CF centre? How quickly can the team be reached? Patient perception of the professional’s consideration for the problem raised? How long does it take to get a response? What types of responses are provided? Scientific recommendations or best practices can be integrated into the wording of questions or response proposals for their scoring: for example, a response provided within 24 h to a telephone call or an e-mail from a patient (maximum score reflecting best practice), or within 2 days, within 1 week or more than a week (scoring = 0). Four versions of the questionnaire will be developed, one for each group. The questionnaire will be designed in order to avoid unnecessary questions and to limit completion time and improve the response rate. For example, if the patient has not been hospitalised in the past 12 months, the questions about their hospitalisation experience will be skipped. For the same purpose, the questionnaire can be administered in different parts.

Construct validation is based on the study participants' responses to the questionnaire. These responses will be put into perspective with the results obtained from interviews conducted with the same participants in Phase 2 of the study in order to assess their fidelity to the patients' or parents' verbatims. The answers will be processed as for a routine use of the questionnaire by consolidating the results by items and categories. The answers to the questions about its usefulness and ease of use will help to suggest improvements to the questionnaire or to its administration process. Finally, a synthesis of the results will be presented to the associated CF centres in videoconferences in order to discuss the usefulness of the questionnaire-based survey in evaluating the quality of care and identifying how it can be used by CF centres or the National CF network.

## Results

### Recruitment of patients and parents to become co-researchers in the research group

Seven patients and parents of children with CF were recruited in January 2020 to join the research group: 2 parents of children aged 4 and 9 years; 2 non-transplanted patients (20 years and 30 years); 3 lung transplant patients at different times since transplantation (2 years, 5 years, 15 years). All were informed about the process of the study and the expected level of participation, and their formal consent to participate was obtained. They alone represent a diversity of health and demographic situations in the cystic fibrosis pathway that has great potential for enriching the thinking process undertaken in the study. The research group thus includes a total of 7 patients and parents, 4 professionals (3 doctors and 1 nurse) and 5 researchers from two laboratories LEPS/USPN and RESHAPE/Lyon 1 University.

### Training of patient and parent co-researchers in qualitative research

The content and stages of training are described in Appendix 2. The objectives are as follows:

- elaborating a protocol: defining participant inclusion criteria, elaborating interview guides based on participant profiles, conducting mock interviews between co-researchers, drafting information and consent forms;
- knowing the regulatory procedures and submitting a protocol for ethics committee approval according to the research methodology used;
- conducting mock semi-structured interviews with an academic researcher;
- performing the thematic analysis of interviews with an academic researcher;
- elaborating and testing PREM questionnaires based on the themes resulting from the analyses;
- administering the questionnaires and processing the patients' and parents' answers;
- validating the construct with the participants and teams of the centres involved in the study.

### Ethics and approval

Ethical approval was granted for this study protocol by the INSERM Ethics Evaluation Committee during the session held on 9th June 2020.

*IRB Agreement no* IRB00003888—Notice n°20-700.

*Issue date*: 9th June 2020.

### Study setting

**Fig. 2:**
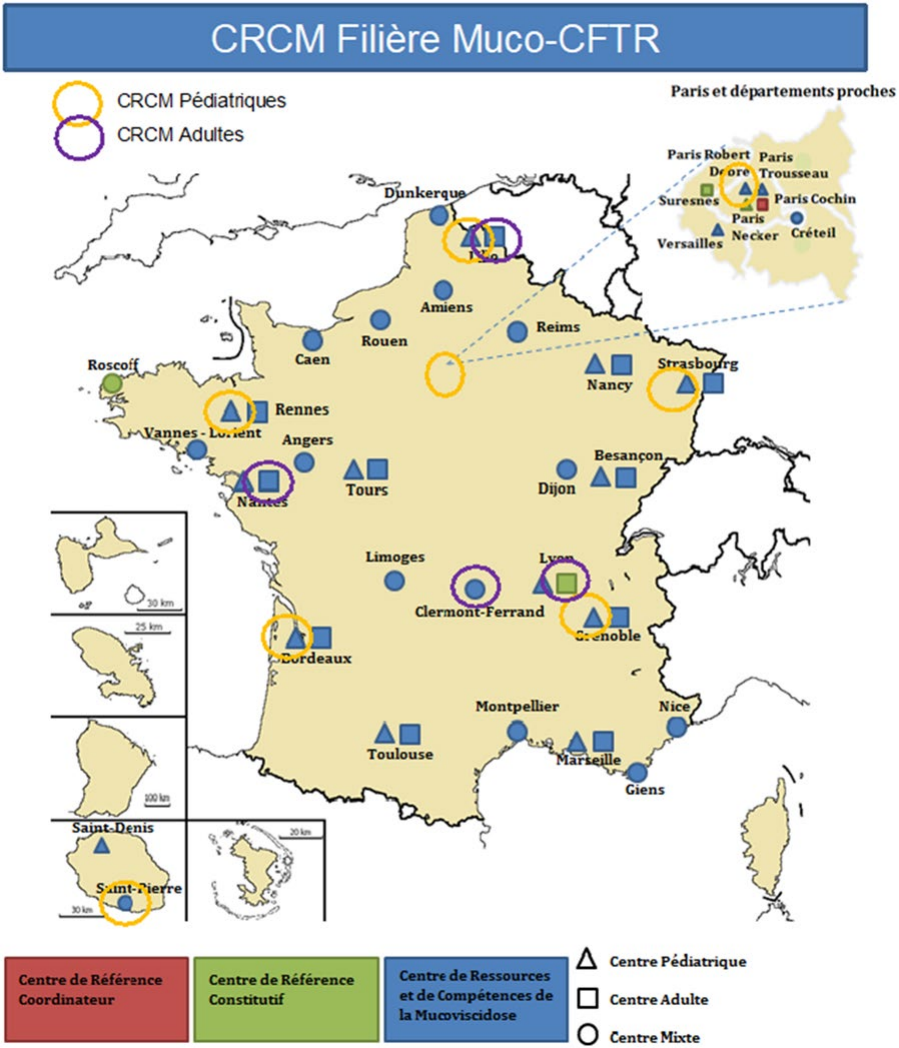
Geographical distribution of associated centres of the study

Associated Paediatric Centres are Bordeaux, Paris R. Debré, Grenoble, Lille, Rennes, Saint-Pierre La Réunion, Strasbourg. Associated Adult Centres are Clermont-Ferrand, Lille, Lyon, Nantes (Fig. 2).

## Discussion

The aim of this study is to develop cystic fibrosis PREMs in the context of collaborative research involving patients and parents, CF centres care professionals, and academic researchers. This study should lead to the development of a common construct that will measure what matters most to patients [42] and be easily used by the actors of the CF network. In recent years, various questionnaires have been developed internationally to assess the quality of CF care from the point of view of the patients. The ExPaParM PREM stands out in the fact that it covers the domains of health, behaviours, and life with CF and it questions the transition points such as the diagnosis of the disease, transition to adult care, and transition to transplant. By focusing on CF care, the instrument reports on specific aspects of the French model of care including out-of-hospital care, patient education, and physiotherapy by out-of-hospital professionals, and the role of local pharmacists where patients live. This project is expected to provide a more comprehensive understanding of patients’ situations and difficulties, in order to suggest areas of improvement for the various actors in the French CF network in terms of care and social conditions for patients and their families.

### An innovative patient partnership in research

The patient-caregiver partnership is used by different groups or dynamics of the French CF network: the national Therapeutic patient education group (GETHEM) since its creation in 2005, the quality improvement programme deployed between 2011 and 2019, the structured committees for the governance of the CF network. The ExPaParM project innovates by working on a patient partnership in a research project that includes patients and parents as co-researchers. This partnership enhances the experiential knowledge of patients and parents and develops their knowledge and understanding of the qualitative scientific methodology. It responds to their wish to improve the patient pathway for the greatest number of their peers. Patients are increasingly involved in different stages or activities of research, from suggesting research themes that are relevant to them, to interpreting data or in the communication of results [43]. In our collaborative research, patient partners are expected to participate in: defining the situations to be investigated and enriching the research tools; conducting interviews; participating in the thematic analysis; co-constructing the PREM questionnaires; interpreting the results and valorising them in conferences and publications; and promoting the project to the relevant authorities. Certain conditions, such as their training, can enhance this participation at all stages of research [44]. Facilitating resources to patient and parent co-researchers’ engagement such as remote communication tools and time compensation are made available for the project. Group discussions between researchers, patients, and parents are planned during the study to share the patients’ perspectives on their engagement and sustain their motivation to participate in the research. This study may contribute to a better understanding of the conditions suitable for patient participation in research and for their individual and collective empowerment through this participation.

### Challenges of this study

As this study builds on a large variety of care and living situations to be investigated, it led to a larger sample of participants than in most qualitative exploratory research, although it is not representative of the frequency of occurrence of these situations in the total population of CF patients in France. As a result, the themes that arise from analysing the interviews cannot be weighted in terms of their occurrence and doing so would not be relevant. All the themes will then be translated into the PREM questionnaire submitted for construct validation among the study sample. This choice, which is inherent to the method used, allows for the collection of patients’ experiences that might seem exceptional. But first, some of these experiences might be more frequent in a larger sample than anticipated. Second, an exceptional situation may highlight a structural problem in the organisation of care (as is the case in the overseas departments), which shows healthcare inequalities between territories. Finally, this method can reveal possible variations in care pathways (post-transplant follow-up) to be looked into in terms of the quality and safety of care. This would help identify best practices in conjunction with the need to personalise the follow-up according to patients' needs.

Subsequent psychometric validation will allow the weighting of the themes and items in a large sample of respondents and to possibly reduce the number of items or adapt the course of the questionnaire to a patient profile, depending on the objectives sought [45].

### Perspectives

In France, the *Haute Autorité de Santé* (HAS) has published a report on international experiences in setting up PREMs and the lessons learned on patients' perception of quality of care [46]. ExPaParM proposes a method to co-construct a PREM based on patient experience from a large number of health and life situations in the context of a rare disease. The journeys of people living with a rare disease have distinctive characteristics beginning from (and sometimes before) the diagnosis, with multiple transitions and a level of complexity of care and life with the disease that can only be reported by the people themselves and their relatives [47]. PREMs are therefore as much a descriptive tool for developing a typology of patient pathways and point out problem areas, as they are a tool for evaluating the quality of intervention of various actors in these pathways.

This tool may be used in many different ways to improve patients’ experience of care and life with the disease: in the context of the individual caregiver-patient relationship, to identify difficulties encountered by the patient in dealing with the disease; at the level of a health care centre, to identify organisational or structural problems, identify groups of patients who are experiencing more difficulties and analyse the impact of changes in practices; at the level of a rare disease network, to benchmark the organisations and practices that contribute to improving patients’ experience; at the system level to assess the impact of therapeutic or technological innovation such as a new treatment or telemedicine; at the level of patient associations, to identify the type of psychosocial support to be offered to groups of patients experiencing difficulties and the areas of advocacy to be developed.

## Conclusion

The ExPaParM project stands out as its method is based on a collaborative and qualitative research approach that focuses on the lived experience of patients along their pathways of care, health, and life with a rare disease. This approach allows for a holistic vision of the patient's journey and their needs, without limiting the themes that are looked into to the sphere of activity of the actors involved in their care. As a result, it can help identify gaps in care or support and encourage the emergence of new interventions or actors. Its descriptive and evaluative nature make it sensitive to changes in care pathways, which is useful in the context of therapeutic or technological innovation.

Internationally, the development of PREMs, including quality scores on common themes of CF pathways, can allow a benchmarking between countries with different health systems and organisations. Additionally, it may foster the interest of the CF community on improving the conditions of daily care and social life beyond hospital care.

## Data Availability

Not applicable.
